# Multi-Output Monitoring of High-Speed Laser Welding State Based on Deep Learning

**DOI:** 10.3390/s21051626

**Published:** 2021-02-26

**Authors:** Boce Xue, Baohua Chang, Dong Du

**Affiliations:** 1 Department of Mechanical Engineering, Tsinghua University, Beijing 100084, China; xbc17@mails.tsinghua.edu.cn (B.X.); bhchang@tsinghua.edu.cn (B.C.); 2 Key Laboratory for Advanced Materials Processing Technology, Ministry of Education, Beijing 100084, China

**Keywords:** laser welding, monitoring, deep learning, multi-output prediction, particle swarm optimization, CNN visualization

## Abstract

In order to ensure the production quality of high-speed laser welding, it is necessary to simultaneously monitor multiple state properties. Monitoring methods combining vision sensing and deep learning models are popular but most models used can only make predictions on single welding state property. In this contribution, we propose a multi-output model based on a lightweight convolutional neural network (CNN) architecture and introduce the particle swarm optimization (PSO) technique to optimize the loss function of the model, to simultaneously monitor multiple state properties of high-speed laser welding of AISI 304 austenitic stainless steel. High-speed imaging is performed to capture images of the melt pool and the dataset is built. Test results of different models show that the proposed model can achieve monitoring of multiple welding state properties accurately and efficiently. In addition, we make an interpretation and discussion on the prediction of the model through a visualization method, which can help to deepen our understanding of the relationship between the melt pool appearance and welding state. The proposed method can not only be applied to the monitoring of high-speed laser welding but also has the potential to be used in other procedures of welding state monitoring.

## 1. Introduction

Laser welding is a complicated manufacturing process with high energy density and high efficiency, while humans have limited understanding of the process mechanism [1,2]. The welding quality can be influenced by various factors such as the internal defects of materials and the complex manufacturing environment. Therefore, monitoring of the laser welding process is essential for making high quality production [3,4].

Since multiple factors influence the appearance of the melt pool, images of the melt pool captured by a vision sensor contain rich information about the welding process [5], and can reflect the condition of the welding process, namely welding state. In addition, compared with other sensors like an acoustic emission sensor and photodiode sensor, a vision sensor has advantages considering the quality of the collected information, the position of the sensor and the industrial application. Therefore, researchers frequently used vision sensors to capture images of the welding process and monitored the welding state [6]. Hand-crafted image processing algorithms were used to extract features from images and machine learning methods like support vector machine [7,8,9,10], random forest [5], and k-nearest neighbors [11] were applied to predict the welding state. However, image data captured by vision sensors can be complex and high-dimensional. Feature extraction methods through hand-crafted image processing algorithms require much prior knowledge and are always specific to the task. Because deep learning methods like convolutional neural network (CNN) rely more on real data and less on hand-crafted feature extraction methods and have a more powerful learning ability, they are more suitable than classical methods for the analysis of complex high-dimensional data like image data [12]. Therefore, researchers started to introduce deep learning to analyze image data in the research of the welding process monitoring in recent years [13]. Zhang et al. [6] captured coaxial images of the weld pool in laser welding and used a CNN for the penetration state diagnosis. Zhang et al. [14,15] built a multiple-sensor system consisting of a spectrometer, two photodiodes and two visual sensors to monitor the laser welding state and used deep learning methods including stacked sparse autoencoder and CNN to model the relationship between the multi-sensor features and their corresponding welding state. Liu et al. [16] combined the CNN and long short-term memory network to realize the mapping from melt pool images to defects in CO_2_ welding. Feng et al. [17] built a framework called DeepWelding, which applies multiple deep learning techniques to achieve better performance in the monitoring of gas tungsten arc welding. Bacioiu et al. [18,19] used CNN models to detect defects in tungsten inert gas welding. Shevchik et al. [20] proposed a method for real-time detection of laser welding instabilities by the application of CNN.

**Figure 1:**
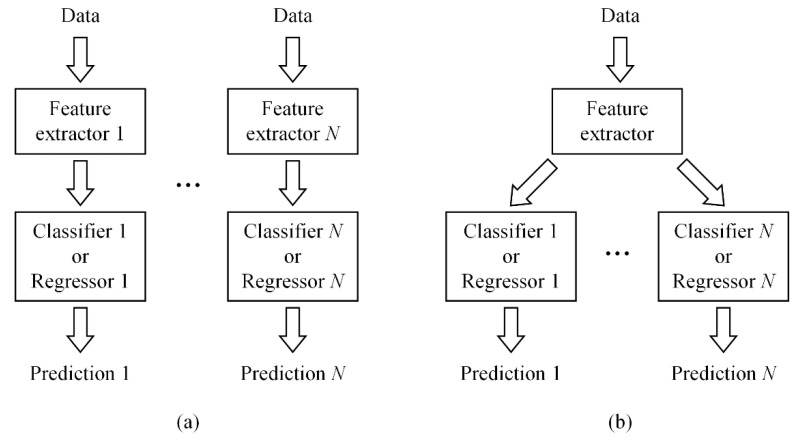
Different forms of multi-output prediction models. (**a**) Model consisting of a series of sub-models. (**b**) Model consisting of a public feature extractor and multiple predictors.

The production efficiency of laser welding can be improved with the improvement of welding speed. Although many defects can occur in laser welding, like spatter and misalignment, humping is one of the most typical defects in high-speed laser welding [21,22]. In addition, because of performance problems of the equipment or the misoperation by workers, the actual welding parameters including laser power, welding speed and defocusing amount may deviate from the required values. For example, the aging of the laser can make the output laser power smaller than the setting power, and a speed sensorless motion device cannot ensure steady welding speed [23,24]. These problems are difficult to find in high-speed laser welding with naked eye as the welding process can be very quick. Under such circumstances, even no obvious defect appears, the performance of the weld can also be substandard. Therefore, in order to achieve comprehensive monitoring of the high-speed laser welding, it is necessary to simultaneously monitor the occurrence of defects and welding parameters. In this research we focus on the occurrence of humping, as well as the values of laser power and welding speed. Generally, the occurrence of humping is related to laser power and welding speed. However, if we predict the laser power and welding speed first and then predict the occurrence of humping according to the predicted values of the laser power and welding speed, the result will be influenced by prediction errors of laser power and welding speed. If all outputs are predicted directly from the input data, the output could be more accurate. Therefore, a multi-output model able to perform classification and regression simultaneously is required. However, in the existing research of welding state monitoring, researchers mainly built CNN-based single-output models to analyze collected image data. To build a multi-output model, one way is to build a series of single-output sub-models to predict each of these properties, respectively, as shown in Figure 1a. However, if the multi-output model is designed in this way, each sub-model needs to extract features from the original data by itself and cannot share features with other sub-models, so the building and using of the model can be tedious and inefficient. In order to simplify the model and improve efficiency, another way is to build a public feature extractor to extract features from the original data, and then input features to several predictors to make prediction of multiple properties, as shown in Figure 1b. 

Based on this idea, in this paper we propose a multi-output model based on CNN, to simultaneously monitor multiple state properties of high-speed laser welding according to images of the melt pool. A lightweight CNN architecture MobileNetV2 is adopted as the public feature extractor, followed by several fully connected layers in parallel to output predictions of multiple properties. What is more, particle swarm optimization (PSO) is introduced to optimize the loss function of the network, and the network achieves better performance after the optimization. Images of melt pool are collected through high-speed imaging of high-speed laser welding of AISI 304 austenitic stainless steel, and the dataset is established. Then the proposed model is applied to simultaneously monitor multiple state properties of high-speed laser welding. Performances of the proposed model and other models are compared and analyzed. In the end, a CNN visualization technique Grad-CAM is applied to interpret the decision of the model, which can deepen our understanding of the melt pool appearance of high-speed laser welding. This paper is organized as follows. Section 2 introduces the architecture of the proposed model. Section 3 presents welding experiments and data processing. Section 4 gives analyses and discussions on test results. Section 5 presents the summary.

## 2. Architecture of the Model 

### 2.1. Multi-Output Model Based on MobileNetV2

**Figure 2:**
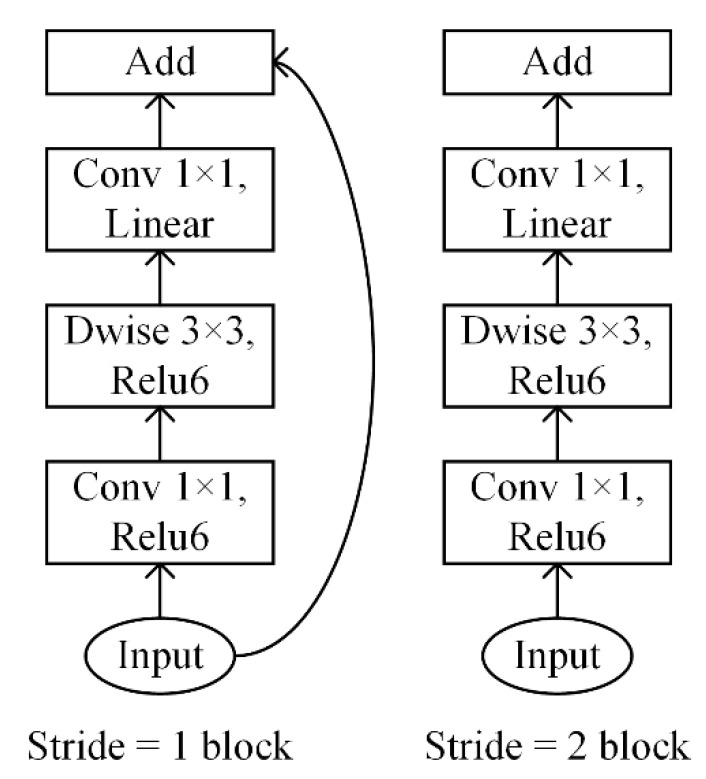
Structure of the building block of MobileNetV2.

The proposed model is based on MobileNetV2 [25], an efficient lightweight CNN architecture, which performs well on ImageNet classification, COCO object detection and VOC image segmentation. Compared with other modern networks, it decreases the number of operations and the memory needed while retaining high accuracy and is especially suitable for resource constrained environments like industrial environments. The basic building block of MobileNetV2 is a bottleneck depth-separable convolution with residuals, whose structure is shown in Figure 2, and it has the following characteristics. First, it replaces the standard convolution operation with the depthwise separable convolution which consists of two separate layers. The first layer is called a depthwise convolution that performs lightweight filtering by applying a single convolutional filter per input channel, and the second layer is called a pointwise convolution that performs 1 × 1 standard convolution. The computational cost of the depthwise separable convolution is much smaller than that of the standard convolutions at only a small reduction in accuracy. Second, the linear bottleneck layer is inserted into the end of the building block to prevent non-linearities from destroying too much information. Third, an inverted residual structure is introduced. The shortcut connection is inserted between the building blocks, as shown for the block with stride = 1 in Figure 2. This design can improve the ability of a gradient to propagate across multiplier layers. In addition, in the building block the input is expanded to high dimension at the start and projected back to low dimension at the end, in order to make it possible for the network to represent more complex functions. The architecture of MobileNetV2 contains the initial fully convolution layer with 32 filters, followed by 19 residual bottleneck layers. What is more, the original MobileNetV2 is aimed at color images with 3 channels, but in this research the input image is grayscale images with a single channel, so the input channel of MobileNetV2 is changed from 3 to 1.

**Figure 3:**
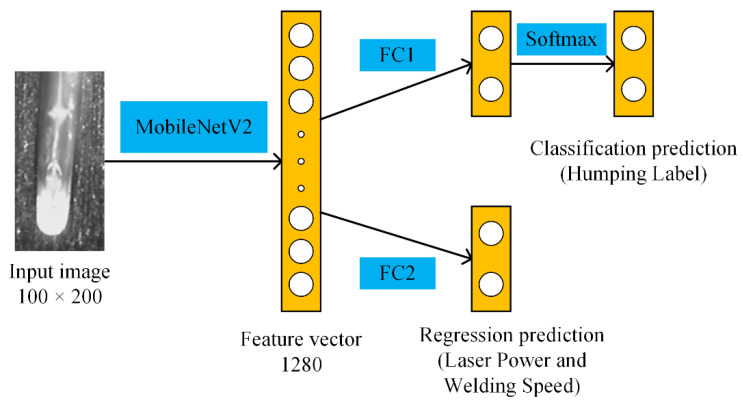
Architecture of MobileNetV2-C&R.

For an input image, MobileNetV2 outputs a feature vector with a length of 1280. In order to achieve the multi-output prediction, we propose the MobileNetV2-C&R network model, where 2 fully connected (FC) layers are added in parallel at the end of MobileNetV2. One FC layer followed by a softmax function is used for the classification prediction, outputting the humping label (humping or no humping). The other is used for the regression prediction, outputting values of laser power and welding speed. The output of the model can be expressed as [humping label, laser power, welding speed]. The architecture of the model is shown in Figure 3. In the training process of the network, a loss function is required. Generally, a common loss function for classification problems is the cross-entropy loss, and for regression problems is the mean square error loss. In MobileNetV2-C&R, both classification and regression are performed, so we build a function $L_{T}$ as the total loss function:

(1) $$L_{T}=10^{A}L_{C}+L_{R}$$

where $L_{C}$ is the cross-entropy loss for classification, $L_{R}$ is the mean square error loss for regression, A is a hyper-parameter used for adjusting the relative weight between $L_{C}$ and $L_{R}$ in $L_{T}$. Because the cross-entropy loss and the mean square error loss may be different in order of magnitude, A is placed in the exponent.

### 2.2. Optimization of the Loss Function

Hyper-parameter A in the total loss function of MobileNetV2-C&R needs to be determined. Loss functions for classification and regression have different forms, so if we simply set A=0, the network may not get trained effectively. Therefore, particle swarm optimization (PSO) [26] is introduced to optimize the value of A, namely MobileNetV2-C&R-PSO model. PSO is an evolutionary computation technique that originates from the simulation of a simplified social model. It comprises a simple concept and is computationally inexpensive, with satisfactory convergence speed and high tolerance to initial parameters and is popular in the optimization of nonlinear functions. Main processes of PSO are as follows:

1. Set the parameters of PSO, including the number of particles n, learning factors $c_{1}$ and $c_{2}$, and the inertia weight w.
2. Randomly initialize the velocity and position for each particle. The velocity is $v_{i}$ and the position is $x_{i}$ for the i-th particle.
3. Calculate the value of the fitness function for each particle.
4. According to the value of the fitness function, determine the best position for each particle in history $pbest_{i}$ and the global best position for all particles in history gbest.
5. Determine whether the maximum iteration is reached. If so, output gbest and finish the optimization. Otherwise update the velocity and position for each particle:
  (2) $$v_{i}=wv_{i}+c_{1}r_{1}\left(pbest_{i}-x_{i}\right)+c_{2}r_{2}\left(gbest_{i}-x_{i}\right)$$
  where $r_{1}$ and $r_{2}$ are stochastic variables between 0 and 1,
  (3) $$x_{i}=x_{i}+v_{i}$$

Then return to step (3).

To achieve satisfactory performance in both classification and regression, the fitness function is designed as follows. Substitute $A=x_{i}$ into Equation (1) and train the network for 1 epoch with the train set, and then test the trained network on the train set. For the test result of classification, we use balanced accuracy for evaluation since numbers of humping samples and no humping samples are different in the following dataset:

(4) $$a_{\mathrm{balanced}}=\frac{a_{h}+a_{nh}}{2}$$

where $a_{h}$ and $a_{nh}$ are classification accuracies on samples with ground-truth labels of humping and no humping, respectively. 

The test result of regression is evaluated with the coefficient of determination $R^{2}$ [5]:

(5) $$R^{2}=1-\frac{\sum_{i=1}^{n}{\left(y_{i}-{\hat{y}}_{i}\right)}^{2}}{\sum_{i=1}^{n}{\left(y_{i}-\bar{y}\right)}^{2}}$$

where n is the number of samples, ${\hat{y}}_{i}$ is the predicted value of the i-th sample, $y_{i}$ is the corresponding true value, $\bar{y}$ is the mean value of $y_{i}$ of all samples. The best possible value of $R^{2}$ is 1.0.

The fitness function combines evaluation indices of both classification and regression:

(6) $$f=a_{\mathrm{balanced}}+\frac{R^{2}{}_{\mathrm{power}}+R^{2}{}_{\mathrm{speed}}}{2}$$

where $R^{2}{}_{\mathrm{power}}$ and $R^{2}{}_{\mathrm{speed}}$ are $R^{2}$ of laser power prediction and welding speed prediction, respectively. 

**Figure 4:**
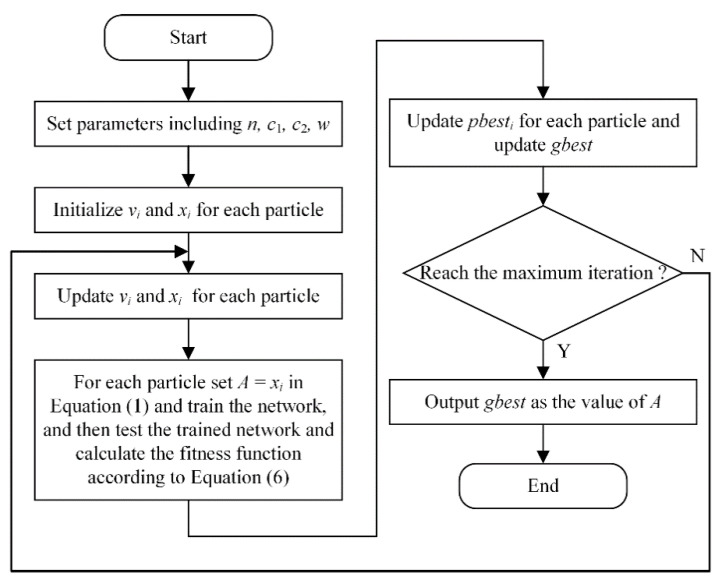
Flowchart of the particle swarm optimization (PSO) optimization.

PSO is applied to search for the optimal value of A which leads to the largest value of f. The flowchart of the optimization process is shown in Figure 4. Then the optimal value of A is substitute into Equation (1) and the loss function of MobileNetV2-C&R-PSO is got.

## 3. Data Acquisition and Processing

### 3.1. Experimental Setup and Procedure

**Figure 5:**
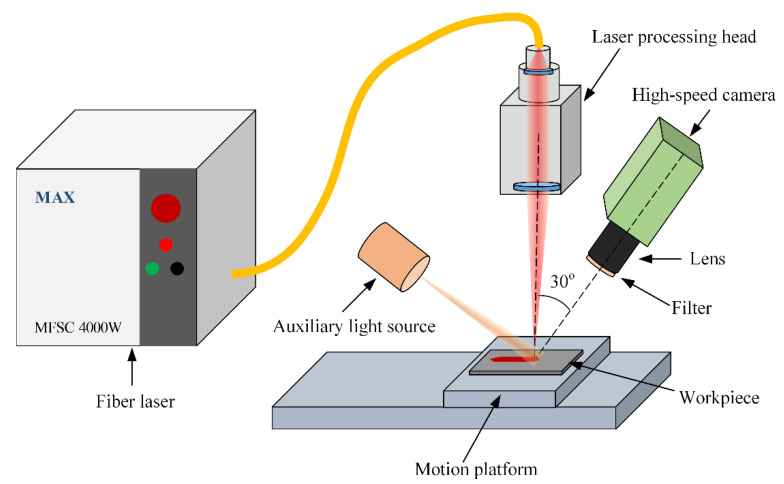
Experimental configuration.

High-speed imaging experiment of high-speed laser welding is conducted to capture images of the melt pool. The welding method is bead-on-plate welding without the application of external filler material. The experimental configuration is shown in Figure 5. Workpieces are AISI 304 austenitic stainless-steel sheets with dimensions of length 200 mm × width 50 mm × thickness 1 mm. AISI 304 austenitic stainless steel is the most popular group of high-alloy stainless steels with high corrosion resistance and good strength [2]. One workpiece is fixed on the base plate of a WN500TA electric motion platform from Winner Optical Instruments during each experiment. The base plate is made of 6061 aluminum alloy and has dimensions of length 300 mm × width 150 mm × thickness 10 mm. Workpieces are cleaned with absolute ethyl alcohol before the welding process. The Argon is used as the shielding gas and supplied on the face side of the workpiece at a flow rate of 20 L/min. A MAX MFSC 4000 W single-mode fiber laser and a Precitec YC52 laser processing head are integrated to perform laser welding. The maximum power of the fiber laser is 4 kW and the wavelength of the laser is 1070 nm. The focal lengths of the collimation lens and focus lens are 150 mm and 300 mm, respectively, and the feeding fiber core diameter is 0.2 mm. The defocusing amount of the laser spot is set to 0. A NAC Memrecam HX-6 high-speed camera is used to record images of the melt pool during the welding process and its frame rate is set to 5 kHz. The camera is placed in front of the melt pool and its axis forms a 30° angle with the vertical direction. A Cavitar CAVIULX HF pulsed high power diode laser light source is used as an active light source for illumination and its central wavelength is 810 nm, and a narrow band-pass filter with the central wavelength of 810 nm is attached on the camera lens. A total of 15 experiments are performed. The laser power ranges from 1.5 kW to 3 kW with step of 0.5 kW and welding speed ranges from 12 m/min to 24 m/min with step of 4 m/min. Visual tests are carried after the welding process to judge whether humping occurs.

### 3.2. Data Preprocessing

Because welding speeds in experiments are high, there exist obvious acceleration and deceleration processes when the motion platform starts and stops. Therefore, 1000 images are selected for each experiment when the welding speed is stable. Then 800 of them are selected randomly and added into the training set, and the rest into the test set. In total the training set contains 12,000 samples and the test set contains 3000 samples.

**Figure 6:**
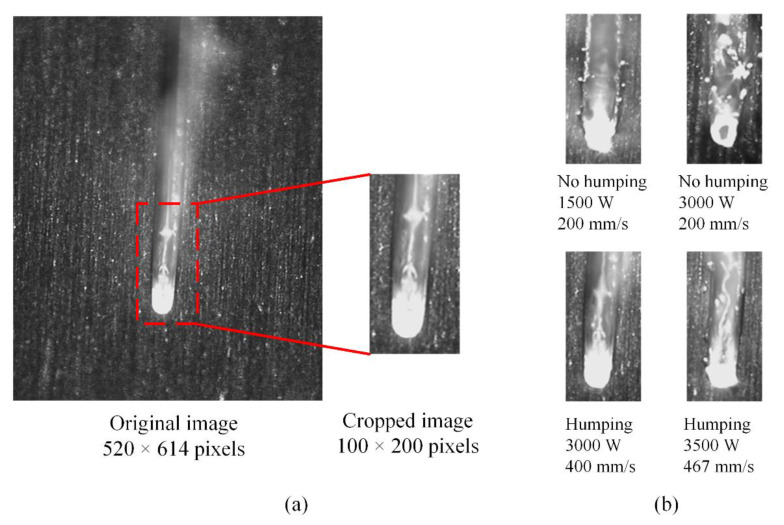
Image cropping. (**a**) Illustration of the cropping process. (**b**) Examples of the cropped images.

For all images in the dataset, image cropping is performed. The cropping process is shown in Figure 6a and examples of cropped images with different welding states are shown in Figure 6b. There are two purposes of image cropping. First, it can shorten the time cost of the training and testing processes. Second, most region irrelevant with the melt pool is removed, so the model can focus more on the melt pool and learn related rules.

**Figure 7:**
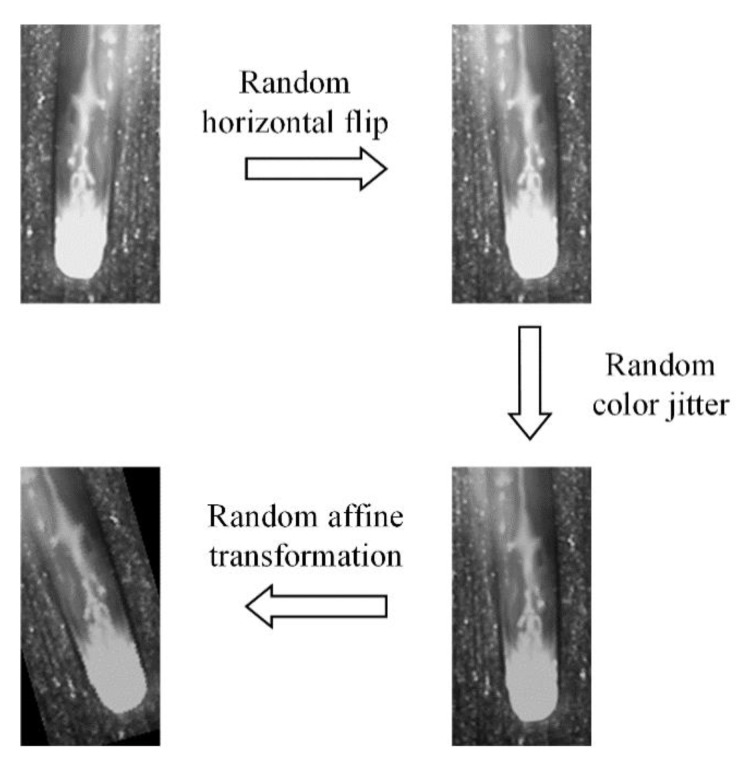
Data augmentation process.

Next, data augmentation is performed for images in the training set, which can enhance the diversity of the data, help to overcome the overfitting in the training and improve the robustness of the trained model [27]. Three transformations are performed in sequence, including random horizontal flip with a probability, random color jitter (random changing of the brightness, contrast and saturation) and random affine transformation. The process is shown in Figure 7.

### 3.3. Training of the Model

**Figure 8:**
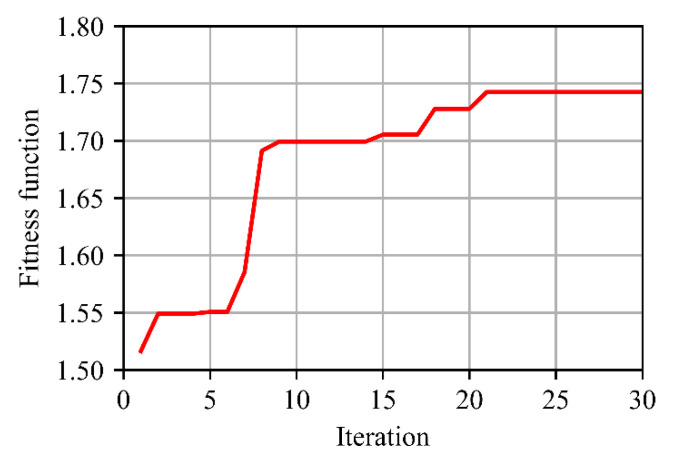
Change of the fitness function in the PSO optimization process.

Based on the preprocessed training set, PSO is introduced to optimize the loss function of the network, as described in Section 2.2. Parameters of PSO, including n, $c_{1}$, $c_{2}$, and w, are set to 5, 0.5, 0.5, 0.8, respectively, and 30 iterations are performed. The change of the fitness function in the optimization process is shown in Figure 8. The value of the fitness function rises to 1.7486 and the corresponding optimal value of A is −0.9347. Then with the optimized loss function, the network is trained for 30 epochs. In the training process, the batch size is 20 and Adam algorithm [28] is implemented. What is more, dropout [29] is used before FC layers to alleviate the overfitting. Finally, we get the trained MobileNetV2-C&R-PSO model.

In order to better evaluate the performance of MobileNetV2-C&R-PSO, we also train other models for comparison. These models are as follows:

1. The original MobileNetV2-C&R model where *A* in the loss function is set to 0.
2. Two individual models based on MobileNetV2, one for classification (MobileNetV2-C) and the other for regression (MobileNetV2-R).
3. Classical models. Two classical image feature operators, histogram of oriented gradient (HOG) [30] and local binary pattern (LBP) [31], are used to extract features in images, respectively. Then two classical machine learning methods, support vector machine (SVM) and k-nearest neighbor (KNN), are used to make predictions. The classification and regression forms of SVM are SVC and SVR, respectively, and of KNN are KNC and KNR, respectively. Therefore, four models are built for classification prediction (HOG + SVC, HOG + KNC, LBP + SVC, LBP + KNC) and four models are built for regression prediction (HOG + SVR, HOG + KNR, LBP + SVR, LBP + KNR).

These models run on a computer with the following configuration: Intel(R) Core(TM) i7-8700 CPU@3.20 GHz, 16.0 GB RAM, and GeForce GTX 1060 6 GB GPU. The GPU is used for accelerating the computation of CNN models. The deep learning models are built with Pytorch and classical models are built with Sklearn.

## 4. Results and Discussions

### 4.1. Performance Evaluation and Comparison 

**Figure 9:**
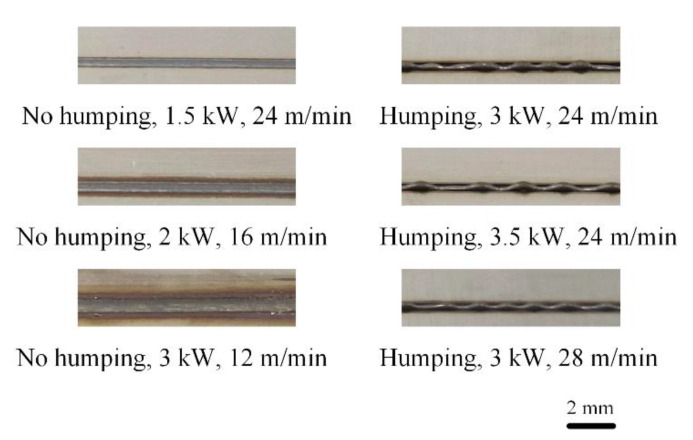
Weld appearance under different welding parameters.

Through visual tests, we find that humping appears in six experiments and no humping occurs in the other nine experiments. Appearances of some welds in experiments are given in Figure 9. 

Models are trained with the training set and then tested on the test set, and performances of them are evaluated. For the classification problem, the balanced accuracy $a_{balanced}$ given in Equation (4) is used as the evaluation index. For the regression problem, besides the coefficient of determination $R^{2}$ given in Equation (5), the mean absolute error MAE is also used as an evaluation index:

(7) $$\mathrm{MAE}=\frac{1}{n}\sum_{i=1}^{n}\left|y_{i}-{\hat{y}}_{i}\right|$$

In addition, for the deep learning-based model, the average prediction time cost per sample is also given. Results are listed in Table 1 and next we perform analyses on them.

**Figure 10:**
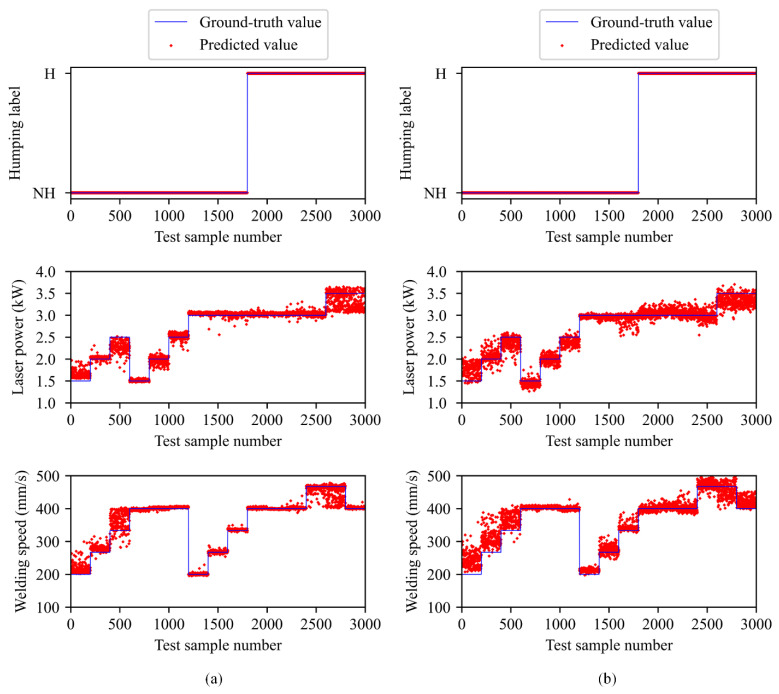
Comparison of the prediction results between (**a**) MobileNetV2-C&R-PSO and (**b**) MobileNetV2-C&R on the test set. (H—humping, NH—no humping).

**Figure 11:**
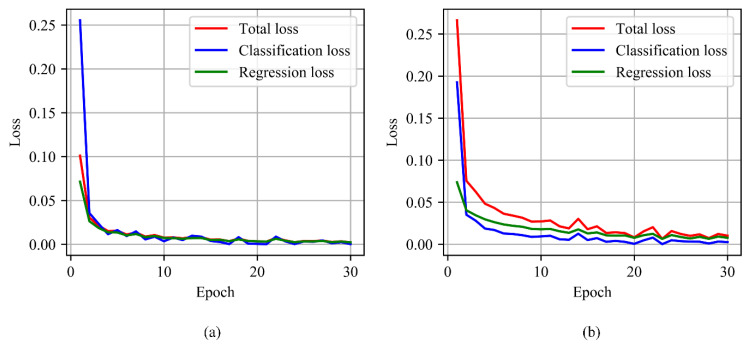
Comparison of loss curves between (**a**) MobileNetV2-C&R-PSO and (**b**) MobileNetV2-C&R in the training process.

First, we compare the performance of MobileNetV2-C&R-PSO and MobileNetV2-C&R. In order to make comparison more intuitively, prediction results of them on the test set are also given in Figure 10. From Table 1 and Figure 10 we can notice that these two models both achieve 100% accuracy on the classification problem. However, MobileNetV2-C&R-PSO performs better on the regression problem, achieving better $R^{2}$ and MAE. In order to explain this difference, loss curves in training processes of these two models are given in Figure 11, from which we can find that classification losses of these two models both converge quickly, but the regression loss of MobileNetV2-C&R-PSO converges obviously faster than that of MobileNetV2-C&R and can converge to a smaller value in the end. In the training process of MobileNetV2-C&R, the classification loss converges faster than the regression loss. In the total loss of MobileNetV2-C&R-PSO, the classification loss has a smaller weight, so in the training process, the network can pay more attention to the regression problem. Therefore, the model can achieve better performance on the regression problem while retaining good classification accuracy.

Next, we compare the performance of MobileNetV2-C&R-PSO, MobileNetV2-C and MobileNetV2-R. From Table 1 it can be observed that on the classification problem MobileNetV2-C&R-PSO and MobileNetV2-C both achieve 100% accuracy, and on the regression problem MobileNetV2-C&R-PSO and MobileNetV2-R also have similar performance. MobileNetV2-C&R-PSO performs slightly better on the prediction of laser power and slightly worse on the prediction of welding speed. However, in terms of the prediction time cost, the combination of MobileNetV2-C and MobileNetV2-R costs 3.73 ms (1.85 ms + 1.88 ms), while MobileNetV2-C&R-PSO only costs 2.24 ms which is 40% shorter. Hence, it can save time while retaining similar prediction performance to replace the combination of MobileNetV2-C and MobileNetV2-R with MobileNetV2-C&R-PSO.

Then we evaluate the performance of classical models. It can be noticed that on the classification problem, models with HOG operator both achieve high classification accuracy, while models with LBP operator perform poorly. On the regression problem, all of these models perform badly. In addition, the performance of these classical models is inferior to that of deep learning-based models. 

Through the above comparison, it can be found that the proposed model MobileNetV2-C&R-PSO is able to perform the monitoring of multiple state properties of high-speed laser welding simultaneously and has satisfactory performance both in terms of accuracy and efficiency. 

### 4.2. CNN Visualization

While CNN based models enable superior performance, their decisions are always thought to be obscure and hard to interpret. In order to explain why CNN based models predict what they predict, several CNN visualization techniques have been proposed in recent years, among which Grad-CAM [32] is a class-discriminative localization technique that generates visual explanations for any CNN based network without requiring architectural changes or re-training. This approach can produce a localization map highlighting the important regions in the image for making decisions. 

For classification problems, in order to obtain the class-discriminative localization map Grad-CAM $L_{Grad-CAM}^{c}$ for any class *c* (humping label in our model), the gradient of the score for class *c*, $y^{c}$ (before the softmax), with respect to feature map activations $A^{k}$ of a convolutional layer (the last convolutional layer of MobileNetV2 in our model) is computed first. These gradients flowing back are global-average-pooled over the width and height dimensions (indexed by *i* and *j*, respectively) to obtain the neuron importance weights $\alpha_{k}^{c}$:

(8) $$\alpha_{k}^{c}=\frac{1}{Z}\underset{i}{\sum }\underset{j}{\sum }\;\frac{\partial y^{c}}{\partial A_{ij}^{k}}$$

Then a weighted combination of forward activation maps is performed, followed by a ReLU to obtain the Grad-CAM:

(9) $$L_{\mathrm{Grad}-\mathrm{CAM}}^{c}=\mathrm{ReLU}\left(\underset{k}{\sum }\alpha_{k}^{c}A^{k}\right)$$

For regression problems, Grad-CAM can also be computed by replacing $y^{c}$ with the predicted regression value $y^{r}$ (laser power or welding speed in our model).

**Figure 12:**
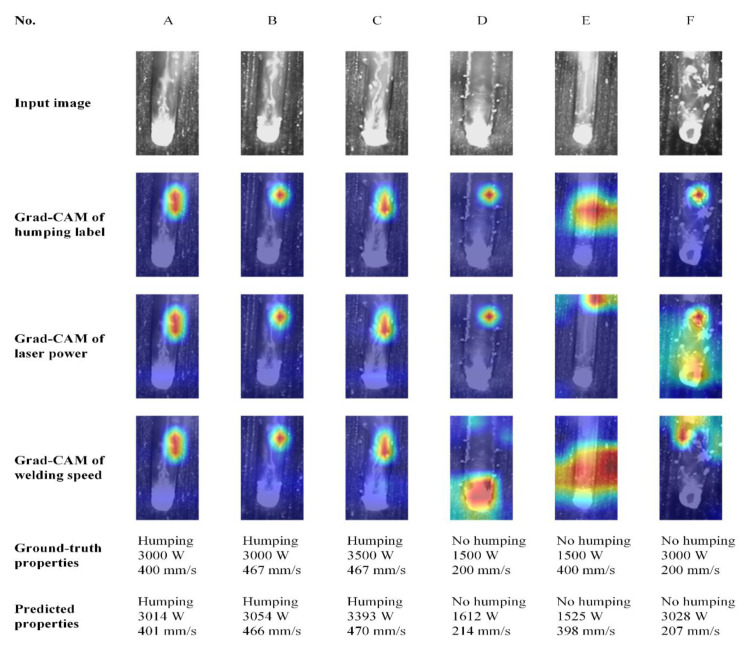
Examples of Grad-CAMs of samples with different welding state.

In order to interpret the prediction of MobileNetV2-C&R-PSO, six samples with different welding states are selected, and their Grad-CAMs of humping label, laser power and welding speed are computed, respectively, as shown in Figure 12. Highlighted red regions in Grad-CAMs are regions having important influence on the prediction of the model, which are mainly concentrated on different parts of the melt pool, indicating that the model makes predictions mainly according to the appearance of the melt pool. Some details are remarkable, which will be discussed next.

**Figure 13:**
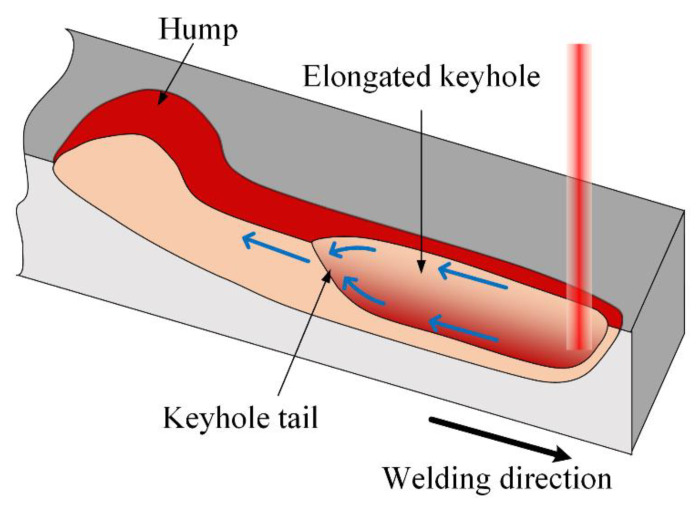
Appearance of the melt pool during humping formation.

In Grad-CAMs of humping label, for all six samples, the highlighted region is mainly concentrated on the melt pool region in the upper part of the image. In fact, the appearance of this region will exactly change once humping occurs. The appearance of the melt pool when humping occurs is shown in Figure 13, which is usually accompanied with high laser power and high welding speed. At this time, the melt stream with high rearward velocity exists in the melt pool, and the instability of the melt stream is the main cause of humping [33,34]. Meanwhile, the keyhole in the melt pool will be elongated, and the liquid level rises at the keyhole tail, which forms a bright region in the image. The highlighted region in the Grad-CAM of humping label is exactly the bright region at the tail of elongated keyhole when humping occurs, which means that the model predicts the occurrence of humping mainly according to the existence this region.

In the Grad-CAMs of laser power and welding speed, for samples A, B, and C, of which the laser power and welding speed are both high, the highlighted region is similar to that in the Grad-CAMs of humping label. This means that the model predicts these samples having large laser power and welding speed also according to the bright region at the tail of elongated keyhole. For sample E with low laser power and high welding speed, the highlighted region in its Grad-CAM of laser power is mainly concentrated on the top of the image. In fact, this sample has the lowest energy density among these samples, so the melt pool is short, and the solidified weld appears on the image top. The diffuse reflection of the light from the auxiliary light source happens here, so this region has high brightness in the image. The model predicts the low laser power of this sample according to the solidified weld. For sample F with high laser power and low welding speed, the highlighted region in its Grad-CAM of laser power is concentrated on the front and middle of the melt pool, which is very different from other samples. In fact, this sample has the highest energy density among these samples, and a penetration hole appears in the keyhole. The model notices the penetration hole and takes it into consideration when predicting the laser power.

To sum up, through the above analysis, it can be found that the model has learned rules and knowledge closely related to the melt pool appearance. This can help us deepen our understanding of the relationship between the melt pool appearance and welding state.

## 5. Summary

In this research, a multi-output model based on deep learning, MobileNetV2-C&R-PSO, is proposed to perform monitoring of multiple state properties during high-speed laser welding of AISI 304 austenitic stainless steel, according to images of melt pool captured by a camera. A lightweight CNN architecture is used to extract features from images, and then two FC layers are added in parallel to solve classification and regression problems simultaneously. In addition, PSO is introduced to optimize the loss function of the model. Dataset is built through the high-speed imaging of high-speed laser welding. By comparing the test results of several models, the proposed model is proven to have satisfactory performance in terms of both accuracy and efficiency. An accuracy of 100% in the prediction of humping occurrence and $R^{2}$ of 0.9570 and 0.9618 in the prediction of laser power and welding speed are achieved, respectively, at an average time cost of 2.24 ms. Then the visualization of the proposed model is performed through Grad-CAM, and the result indicates that the model has learned rules closely related to the appearance of the melt pool. What is more, the architecture of our proposed model is convenient for modification and expansion. By adding more FC layers in parallel, the prediction of more state properties can be achieved, so it also has the potential to be used in other procedures of welding state monitoring. 

## Figures and Tables

**Table 1:** Performance of different models on the test set.

Model		Classification	Regression				Average Prediction Time (ms)
		$a_{\mathrm{balanced}}\;(\%)$	$R^{2}{}_{\mathrm{power}}$	$R^{2}{}_{\mathrm{speed}}$	$MAE_{\mathrm{power}}\;(W)$	$MAE_{\mathrm{speed}}\;(\mathrm{mm}/s)$	
Deep learning based model	Mobile NetV2-C&R-PSO	100.00	0.9570	0.9618	83.17	8.48	2.24
	MobileNetV2-C&R	100.00	0.9416	0.9091	107.83	16.78	2.24
	MobileNetV2-C	100.00	-	-	-	-	1.85
	MobileNetV2-R	-	0.9464	0.9806	92.63	5.84	1.88
Classical model	HOG+SVC	98.01	-	-	-	-	-
	HOG+KNC	94.81	-	-	-	-	-
	LBP+SVC	69.43	-	-	-	-	-
	LBP+KNC	69.28	-	-	-	-	-
	HOG+SVR	-	0.4400	0.5206	369.65	45.40	-
	HOG+KNR	-	0.5463	0.0758	294.33	47.02	-
	LBP+SVR	-	−0.2350	−1.3134	548.53	99.88	-
	LBP+KNR	-	0.0100	−0.1075	414.23	61.42	-
